# Use of reproductive health care services among urban migrant women in Bangladesh

**DOI:** 10.1186/s12905-016-0296-4

**Published:** 2016-03-09

**Authors:** Mohammad Mainul Islam, Anita J. Gagnon

**Affiliations:** Department of Population Sciences, University of Dhaka, Dhaka, 1000 Bangladesh; Ingram School of Nursing, McGill University, Montreal, QC Canada; Research Institute of the McGill University Health Centre, Montreal, QC Canada

**Keywords:** Reproductive health, Care services, Urban, Migrant women, Bangladesh

## Abstract

**Background:**

Recent internal migration flows from rural to urban areas pose challenges to women using reproductive health care services in their migratory destinations. No studies were found which examined the relationship between migration, migration-associated indicators and reproductive health care services in Bangladesh.

**Methods:**

We analyzed the *2006 Bangladesh Urban Health Survey* (data made publically available in June 2013) of 14,191 ever-married women aged 10–59 years. Cross tabulations and logistic regression were conducted.

**Results:**

Migrants and non-migrants did not differ significantly in their use of modern contraceptives and treatment for STI but were less likely to receive ANC even after controlling for a range of variables. Compared to non-migrants, more migrants had home births, did not take vitamin A after delivery, and had no medical exam post-birth. Migrant women being village-born (rather than urban-born) were associated with risk of diminished: use of ANC; treatment for STI; medical exam post-birth; vitamin A post-birth. Migrating for work or education (rather than other reasons) was associated with risk of diminished: use of ANC; use of modern facilities for birth; and medical exam post-birth. Each additional year lived in urban areas was associated with a greater likelihood of receiving ANC.

**Conclusions:**

Women who migrated to urban areas in Bangladesh were significantly less likely than non-migrants to use reproductive health care services related to pregnancy care. Pro-actively identifying migrant women, especially those who originated from villages or migrated for work or education may be warranted to ensure optimal use of pregnancy-related services.

## Background

Rapid urbanization has occurred throughout low-income countries, where 80 % of the world’s largest cities are now located [1]. Such rapid urban growth largely manifests itself in the expansion of already crowded squatter settlements and slums, placing enormous strain on public resources and presenting challenges for local health authorities [2, 3]. The UN Population Division predicts a 93 % increase in the urban population of Bangladesh between 2000 and 2020 driven primarily by rural to urban migration [2, 4, 5]. These recent migration flows pose challenges to women needing reproductive health care services in their migratory destinations. Women in poor countries tend to have limited access to health care services compared to those in richer countries, and within countries, the poor have proportionately less access to health services [6]. Poor migrant women in particular, likely face discrimination as well as physical and economic insecurity, which makes them more vulnerable to reproductive health risks [7].

Studies of rural-to-urban migration often focus on the potential negative effects, physically or mentally, not only of the stress of movement and adaptation to a new and very different lifestyle, but of movement to an area with more health damaging exposures [8]. Damaging exposures may include physical and socio-cultural environments and health behaviours.

Literature on migration shows that use of health and social services for migrants is determined by their legal status and by availability, accessibility, acceptability and quality of services which depend on other influences including social, cultural, structural, linguistic, gender, financial and geographical factors [6, 9–11]. Different beliefs and knowledge about health and illness can deter migrants from using national health services [12]. Moreover, health literacy in the sense of awareness of entitlements to care and availability of services may pose a barrier to use of services [12].

Studies have examined the relationship between migration and general health status, health vulnerability among temporary migrants, rural-urban return migration and use of family planning, urban migrants and under-five child mortality, internal migration and contraceptive knowledge and use, temporary migration and HIV/STD risks, and internal migration and use of reproductive and child health services [13–20]. These studies show that health/reproductive health can be a driver or a barrier to migration through direct and indirect impacts on migration decisions [21]. Studies on the impact of migration on reproductive health show that urban migrants are disadvantaged in accessing health care services [16, 19, 22, 23].

In Bangladesh, studies on migration have focused on reasons for migration, basic characteristics of migrants, employment and income, and household living conditions [24, 25]. Some have focused on the relationship between migration and fertility and family planning, child survival, and household living conditions [17, 26]. These studies have generally used small samples, which have made it difficult to analyze the relationships between key factors comprehensively. No studies were found which examined the relationship between migration, migration-associated indicators and reproductive health care services in Bangladesh. Our study intended to begin to fill this gap.

The objectives of our study were to answer the following research questions: (1) What is the relationship between migration (internal migrant/non-migrant) and Bangladeshi urban women’s use of reproductive health care services [modern contraceptive use, antenatal care (ANC), modern facilities used for birth, medical exam post-birth, Vitamin A post-birth and STI treatment]? (2) What is the relationship between migration indicators (place of birth, length of time living in current place of residence, reasons for migration) and use of these services among internal migrant women in urban Bangladesh?

## Methods

**Fig. 1:**
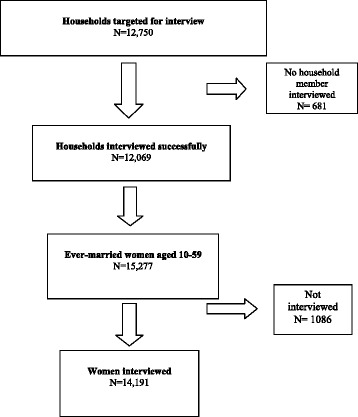
Flow chart 1: Sample taken from the 2006 Bangladesh Urban Health Survey^1^. ^1^National Institute of Population Research and Training (NIPORT), Measure Evaluation, International Centre for Diarrhoeal Disease Research, Bangladesh (ICDDR,B) and Association for Community and Population research(ACPR). 2006 Bangladesh Urban Health Survey. Dhaka: Bangladesh and Chapel Hill, NC, USA, 2008

We performed analyses of cross-sectional survey data from the *2006 Bangladesh Urban Health Survey (UHS)* made publically available on June 24, 2013 [27, 28]. Our sample of 14,191 women was drawn from the sample of all ever-married women aged 10–59 living in separate households in urban areas of Bangladesh (see Fig. 1).

We defined ‘migrant’ as those who had not always lived in their current location. We performed logistic regression analyses of factors affecting selected reproductive health care services. Reproductive health care services selected included modern contraceptive use, ANC received prior to last birth, modern facilities used at birth, medical exam post-birth, receiving Vitamin A post-birth and treatment sought for STIs.

The study protocol was approved by the Institutional Review Board (IRB) of the Faculty of Medicine of McGill University, Montreal, Quebec, Canada. We obtained our data following approval from the University of North Carolina (UNC). Data were de-nominalized by UNC prior to use. All respondents had provided verbal informed consent to be interviewed prior to completing the *2006 Bangladesh UHS*.

## Results

### Relationship between migrant status (migrant/non-migrant) of women and their use of reproductive health care services

Table 1 shows the socio-demographic characteristics of Bangladeshi urban women by migration status (*n* = 8921 migrants, 5270 non-migrants). Compared with non-migrants, more migrant women (*p* = .000 for all comparisons) were aged 19–49 (85.2 vs. 84.2 %), were currently married (83.1 vs. 71.9 %), were married as children (72.2 vs. 67.9 %), had not attended school (36.7 vs. 27.5 %), were living in urban slums (51.5 vs. 41.9 %), had migrated from villages (83.9 vs.11.8 %), and lived in urban locations of Dhaka (44.0 vs. 30.5 %), and Chittagong (26.8 vs. 30.4 %) divisions.

**Table 1:** Selected characteristics of Bangladeshi urban women by migrant status (%)

Characteristics	Migrant (*N* = 8921)	Non-migrant (*N* = 5270)
Age*		
≤18	7.4	8.9
19–24	24.5	28.5
25–29	16.5	17.0
30–34	14.5	13.4
35–39	13.3	11.6
40–44	10.4	8.9
45–49	6.0	4.8
50–54	5.1	4.8
55–59	2.2	2.1
Marital status*		
Currently married	83.1	71.9
Separated/deserted/widowed/divorced	10.4	10.3
Never married	6.5	18.1
Age at first marriage <18 years*	*N* = 8343	*N* = 4317
	72.2	67.9
Not attended school*	36.7	27.5
Muslim Religion**	90.1	91.8
Not working currently*	67.2	76.4
Division (Administrative regions in descending order of density of health care facilities)* ^*a*^		
Dhaka	44.0	30.5
No. of all secondary & tertiary		
hospitals = 40, no. of public hospital		
beds = 6655; population/bed = 6900		
Rajshahi	7.4	15.1
No. of all secondary & tertiary		
hospitals = 26, no. of public hospital		
beds = 4650; population/bed = 7621		
Chittagong	26.8	30.4
No. of all secondary & tertiary		
hospitals = 17, no. of public hospital		
beds = 3550; population/bed = 8002		
Khulna	12.9	14.1
No. all secondary & tertiary		
hospitals = 16, no. of public hospital		
beds = 2015; population/bed = 8536		
Sylhet	4.8	3.1
No. of all secondary & tertiary		
hospitals = 10, no. of public hospital		
beds = 1826; population/bed = 5093		
Barisal	4.2	6.7
No. of all secondary & tertiary		
hospitals = 08, no. of public hospital		
beds = 1370; population/bed = 7010		
Place of current residence*		
Urban slum	51.5	41.9
Urban non-slum	38.1	40.7
District town	10.4	17.3
Origin of birth*		
City corporation	2.9	63.7
District town	9.2	22.8
Other town	2.7	1.0
Village	83.9	11.8^b^
Abroad	1.3	0.6
Illness and healthcare decision making		
Experience of any serious illness in last year*	21.4	18.0
Person who makes final decisions about health care*	*N* = 7688	*N* = 3955
Respondent	24.3	26.8
Spouse	33.5	29.3
Respondent and spouse jointly	32.1	29.6
Someone else	4.5	6.5
Respondent and someone else jointly	5.6	7.8
Perceived security of area*		
Very safe	40.2	46.4
Somewhat safe	55.5	49.0
Unsafe & very unsafe	4.4	4.6
STI symptoms**		
Itching in vaginal area	14.8	13.0
Genital soreness	3.7	2.7
Sexual violence		
Forced to have sexual intercourse	*N* = 7412	*N* = 3790
	19.3	19.9

^a^Government of Bangladesh, DGHS, Health Bulletin 2012: Secondary and tertiary level health care facilities in Bangladesh

^b^Possible explanations: (i) permanently living in cities but born in villages to get support from relatives staying at villages by mother; (ii) This may be due to definitional constraints of ‘urban’. Current place of urban residence might have been regarded as a village at the time respondents were born. For this we examined the length of years lived by both migrant and non-migrant women in the current place of residence considering the current age of the respondents

*Group comparisons by *χ* ^2^, p = 0.000; ** p ≤ 0.02

Migrant and non-migrants differed in terms of RH care services received (Table 2). Compared to non-migrants, more migrant women (p = .000 for all comparisons): did not receive ANC during their last pregnancy (29.3 vs. 15.1 %), had home births more frequently (75.8 vs. 63.5 %), did not have a medical exam post-birth (70.3 vs 57.9 %), and did not take vitamin A post-birth (71.0 vs 65.2 %). Treatment sought for STI symptoms within the last 6 months was lower for migrants (40.9 vs. 44.6 %, *p* = .003).

**Table 2:** Reproductive health care services of Bangladeshi urban women by migrant status (%)

RH care services	Migrant (*N* = 8921)	Non-migrant (*N* = 5270)
*Family Planning*		
Any modern method of family planning (Pill, condom, injection, IUD, female sterilization, male sterilization, implant)	*N* = 4193	*N* = 2157
	88.2	89.5
Sources of current method**	*N* = 3697	*N* = 1930
Public sector: Govt. services	21.5	24.2
NGOs	18.6	18.2
Private medical: hospital/clinic, pharmacy, qualified doctor, traditional doctor	3.5	3.6
Shop, relative/friend	51.3	51.7
Others	6.0	3.9
Don’t know	2.6	2.0
No antenatal care (ANC) at last pregnancy*	*N* = 3421	*N* = 1762
	29.3	15.1
Place of delivery*	*N* = 3421	*N* = 1762
Home (own, parents, in-laws, others)	75.8	63.5
Govt. (hospital, upzila health complex, MCWC-maternal and child welfare centers)	11.1	16.6
Private clinic & NGO	12.9	19.9
Other	0.2	0.0
No medical check after birth*	70.3	57.9
Mother not taken Vitamin A after delivery*	71.0	65.2
Treatment sought for STIs within last 6 months** ^*a*^	*N* = 2595	*N* = 1335
	40.9 %	44.6 %

*Group comparisons by *χ* ^2^, *p* = .000; **Group comparisons by *χ* ^2^, *p* ≤ 0.03

^a^The frequencies of women who sought treatment for STIs in this table account for seven symptoms listed in the survey, and are therefore greater than the frequencies in Table 1, which account for only two symptoms

Table 3 shows the association of migration status (migrant/non-migrant) with reproductive health care services controlling for a range of variables. Migrants and non-migrants did not differ significantly in their use of modern contraceptives and treatment for STI symptoms. However, compared to non-migrants, migrants continued to be less likely to receive ANC [OR = 0.48 (95 % CI 0.41, 0.56)], to give birth in modern facilities [OR = 0.58 (0.50, 0.67)], to not have a medical exam post-birth [OR = 0.63 (0.55, 0.71)], and to take vitamin A post-birth [OR = 0.83 (0.73, 0.94)].

**Table 3:** Association of migration status with reproductive health care services used controlling for group differences (*N* = 14,191)

Variables	Coefficient	Odds ratio	95 % C.I.		Level of Sig.
			Lower	Upper	
Current use of modern contraceptives by married women (*N* = 6350^a^)^b^					
Migrant women	−.12	.89	.75	1.06	.190
ANC used at last pregnancy (*N* = 5183^a^)^b^					
Migrant women	−.73	.48	.41	.56	.000
Treatment sought for STIs (*N* = 3930^a^)^c^					
Migrant women	−.09	.91	.795	1.05	.193
Modern facilities used for birth (*N* = 5183^a^)^b^					
Migrant women	−.55	.58	.50	.67	.000
Medical check after birth (*N* = 5183^a^)^b^					
Migrant women	−.47	.63	.55	.71	.000
Vitamin A after birth (*N* = 5183^a^)^b^					
Migrant women	−.19	.83	.73	.94	.004

^a^Lower than *N* = 14,191 due to missing data; of all women aged 10–59 years, 7.3 % (of 14,191) were past the reproductive span (50–59 years), therefore use of reproductive health care services might not be applicable to this age group

^b^Controlling for current age, Muslim religion, working currently, attended school, slum residence, health care decision by the respondents with or without other, perceived security of the area

^c^Controlling for current age, Muslim religion, working currently, attended school, urban slum as place of current residence

### Relationship between migration indicators and use of reproductive health services among internal migrants to urban areas in Bangladesh

Table 4 shows migration indicators associated with use of reproductive health care services among migrant women only, controlling for a range of other variables. Being village-born (rather than urban-born) was significantly associated with less likely: use of ANC [OR 0.73 (0.57, 0.93)], treatment of STI symptoms [OR 0.75 (0.60, 0.93)], medical exam post-birth [OR 0.65 (0.53, 0.81)], and vitamin A post-birth [OR 0.70 (0.57, 0.85)]. Migrating for work or education (rather than any other reasons) was responsible for less likely: use of ANC [OR 0.83 (0.69, 0.99)], use of modern facilities for birth [OR 0.75 (0.59, 0.95)], and receiving a medical exam post-birth [OR 0.67 (0.54, 0.82)]. Years lived in urban areas by the respondent had a significant beneficial effect on ANC only [OR 1.04 (1.02, 1.06)].

**Table 4:** Factors associated with use of reproductive health care services among migrant women only (*N* = 8921)

Variables	Coefficient	Odds ratio	95 % C.I.		Level of Sig.
			Lower	Upper	
Currently modern contraception used by married women (*N* = 4193^a^)^b^					
Length of years lived	−.01	.99	.98	1.01	.233
Born in villages	.01	1.14	.88	1.46	.322
Migrated for work, employment &education	−.17	.84	.67	1.06	.148
ANC attended during last pregnancy (*N* = 3421^a^)^b^					
Length of years lived	.04	1.04	1.02	1.06	.000
Born in villages	−.32	.73	.57	.93	.010
Migrated for work, employment & education	−.19	.83	.69	1.00	.047
Treatment sought for STIs (*N* = 2595^a^)^c^					
Length of years lived	.00	1.00	1.00	1.00	.099
Born in villages	−.29	.75	.59	.93	.011
Migrated for work, employment &education	.14	1.15	.96	1.39	.140
Modern facilities used for delivery of birth (*N* = 3421^a^)^b^					
Length of years lived	−.01	.99	.98	1.01	.439
Born in villages	−.45	.64	.51	.80	.000
Migrated for work, employment & education	−.29	.75	.59	.95	.016
Medical check after birth (*N* = 3421^a^)^b^					
Length of years lived	−.00	1.00	.98	1.01	.844
Born in villages	−.43	.65	.53	.81	.000
Migrated for work, employment & education	−.41	.67	.54	.82	.000
Vitamin A after birth (*N* = 3421^a^)^b^					
Length of years lived	.01	1.01	1.00	1.03	.088
Born in villages	−.36	.70	.57	.85	.000
Migrated for work, employment & education	−.14	.87	.72	1.06	.167

^a^N is less than 8921 due to missing data; of all women aged 10–59 years, 7.3 % (of 14,191) were past the reproductive span (50–59 years), therefore use of reproductive health care services might not be applicable to this age group

^b^Controlling for length of years lived, born in villages, migrated for work, employment and education, current age, Muslim religion, working currently, attended school, urban slum as place of current residence, healthcare decision by somehow respondent, and safe community security

^c^Controlling for Length of months lived, born in villages, migrated for work, employment and education, current age, Muslim religion, working currently, attended school, urban slum as place of current residence

## Discussion

Bangladesh is facing a rapid increase in its urban migrant population. This poses significant challenges for the country to respond to their health service needs. Difficulties in reaching migrant women to offer reproductive health services could become a barrier in the country’s progress towards the Millennium Development Goals (MDGs). To date, studies on migration in Bangladesh have focused mainly on reasons for migration and basic characteristics of migrants. Some have examined the relationship between migration and fertility and family planning, child survival, and household living conditions [17, 26]. Ours is the first study known to us which looks at the relationship between migration and use of reproductive health care services.

Our study demonstrates that there are significant differences in use of reproductive health care services between urban migrant and non-migrant women in Bangladesh. We found urban migrant women to be vulnerable given their more limited use of reproductive health care services than non-migrants, with the exception of using modern contraception, which migrant women living in slums were more likely to do. Recently (October 14, 2014) disseminated limited preliminary findings of the 2013 *Bangladesh UHS* showed: (i) the contraception prevalence rate to be highest in urban slums and lowest in non-slums; (ii) only half of the women living in slums received ANC from medically trained providers; and (iii) very few home deliveries were attended by medically trained providers in three urban locations – city corporation slum, non-slum and other urban [29]. Our study shows that there are virtually no differences for use of modern contraception among migrant and non-migrant women. This finding might be attributed to easy access to contraceptives, family-planning awareness raised by non-governmental organizations, and a lack of space in urban housing. On the other hand, migrant women reported seeking treatment for STI symptoms less than non-migrant women, although less than half of each group sought care. It is possible that women experiencing symptoms of STIs might think that they need not seek medical attention due to a lack of knowledge and/or the stigma attached to STIs. Thus, policy makers should implement measures to provide both migrant and non-migrant women with equal but increased access to health care services as well as sexual health education.

Of the three migrant indicators examined, being village-born (rather than urban-born) was significantly associated with less use of health services in four of six services examined; migrating for work or education (rather than any other reasons) was responsible for less health care use in three of the six services examined. However, years lived in urban areas had a significant beneficial effect on ANC. This increase in use may be the result of greater exposure to other women who use the service or to messages about the benefits of ANC. On the other hand, lower use of other pregnancy-related services may be the result of a limited supply of health services including maternal health outreach programs for women originating from rural areas. Social prejudice against modern and established health facilities may also contribute to the lower use of such services.

The findings of our study may be applicable to countries with a similar economic profile to Bangladesh such as Cambodia, Kenya, or Cameroon. The average amount of disposable income available to persons residing in these countries may explain differences in access to and use of reproductive health care services. In addition, poverty often forces people to move in search of work and leads to rural-to-urban migration [30]. Expanded trade, and climate change are also driving increased mobility in these countries.

### Strengths and limitations

Our study is drawn from a large and representative data set covering all urban areas in Bangladesh, the result of the first survey of its kind on urban health in Bangladesh. We explored a range of major reproductive health service components as they relate to migration indicators, a field of inquiry previously underexplored in Bangladesh. However, our study was not exempt from limitations, the most important of which was the use of existing data rather than gathering primary data in which questions more closely related to our research questions could have been asked of participants.

## Conclusions

Bangladeshi women who migrated to urban areas in Bangladesh were significantly less likely than non-migrants to use reproductive health care services related to pregnancy care: ANC; giving birth in modern facilities; receiving a medical exam post-birth; and receiving vitamin A post-birth. These women also tended towards less use of modern family planning methods and treatment for STI symptoms, compared to non-migrants. However, migrant women who had lived in urban areas longer were more likely to use ANC. The reproductive health of urban migrant women is under-studied in Bangladesh; data are needed to create and adjust programs to improve their use of health care services. The government of Bangladesh may consider more strategies to address reproductive health care services for urban migrant women. These could include ensuring the availability of services in urban areas for migrant women, especially those who originated in villages and migrated for work and education. It is expected that results from this study will assist in future planning of health and social interventions, internal migration policies, and in defining future research agendas.

## Abbreviations

ANC: Antenatal Care
HIV/AIDS: Human Immunodeficiency Virus Infection/Acquired Immune Deficiency Syndrome
STI: Sexually Transmitted Infections
UHS: Urban Health Survey
UNC: University of North Carolina
